# Contact time has limited impact on the efficacy of disinfectant towelettes when tested under conditions reflective of realistic use

**DOI:** 10.1186/s13756-023-01266-4

**Published:** 2023-07-16

**Authors:** Alyssa M. Kelley, Maxwell G. Voorn, Geraldine M. Tembo, Connor M. Horn, Xiaobao Li, Peter J. Teska, Haley F. Oliver

**Affiliations:** 1 Department of Food Science, Purdue University, 745 Agriculture Mall Drive, West Lafayette, IN 47907 USA; 2 Diversey Inc, Fort Mill, SC 29708 USA

**Keywords:** *Staphylococcus aureus*, *Pseudomonas aeruginosa*, Contact time, Towelettes, Realistic use

## Abstract

**Background:**

Disinfectant towelettes are increasingly being used as a means to prevent transmission of clinically important pathogens which could lead to healthcare-associated infections (HAIs). However, the efficacy of disinfectant towelette products when tested under realistic use conditions is understudied. A test model was designed to replicate realistic wiping conditions. The objective of this study was to determine the impact of varied contact time on disinfectant towelette efficacy under these conditions.

**Methods:**

Five product types were tested against *Staphylococcus aureus* (ATCC 6538) and *Pseudomonas aeruginosa* (ATCC 15,442) at five contact times (30 s, one min, two min, three min, and 10 min) on hard, non-porous laminate templates to determine the impact of contact time on disinfectant towelette efficacy when tested under realistic use.

**Results:**

Product type had a significant impact on the efficacy of disinfectant towelettes when tested under conditions reflective of realistic use. The effect of contact time was limited and no differences in efficacy were seen at a contact time of one min compared with the other contact times tested. Only one disinfectant towelette product achieved a mean 5-log reduction under the tested conditions.

**Conclusion:**

Efficacy of disinfectant towelettes was primarily impacted by product type when applied in a model designed to replicate realistic use in which only a limited effect of contact time was observed. There is a need for further investigation into which factors have the greatest impact on disinfectant towelette efficacy when applied in clinical settings.

## Highlights

Contact time had limited impact on the efficacy of disinfectant towelettes.

Product type significantly impacted the efficacy of disinfectant towelettes.

Disinfectant towelettes did not achieve expected log reductions under conditions of realistic use.

## Introduction

In the United States, an estimated 3.2% of hospital patients are affected by healthcare-associated infections (HAIs) [1]. These infections are caused by several pathogens such as *Staphylococcus aureus* and *Pseudomonas aeruginosa*, both of which ranked among the top four most frequently reported HAI pathogens from 2015 to 2017 [2]. Research suggests that over half of HAIs may be preventable [3]. Cleaning and disinfection of environmental surfaces are often recommended as strategies for controlling HAIs [4] as there is evidence that these measures are associated with lower transmission and infection frequency [5–7]. Improvements in disinfection are of particular interest, as they have been noted to reduce HAI [8] and several studies have indicated that the use of disinfectants results in better outcomes than cleaning alone [9, 10].

In recent years, disinfectant towelette product use has increased [11, 12] as these products provide numerous benefits over traditional methods of disinfection, as reviewed by Boyce, (2021) [13]. Disinfectant towelette products have been demonstrated to out-perform other methods of disinfection [14] and lead to increases in compliance with cleaning protocols [15]. Despite their benefits, experts are concerned that the methods currently used to test products do not accurately reflect the way these products are typically used [16, 17]. There is a need for further research on the performance of disinfectant towelette products under conditions encountered under realistic use, as reviewed by Boyce, 2021 [13] and Song et al., 2019 [11]. This includes the need for further study of realistic contact times [13].

The contact times for many disinfectant products have been criticized as being unrealistic [12]. Berendt et al., (2011) [18] observed wiping to occur for only 1–2 s for a given object. A contact time of one min has been utilized previously to reflect a realistic contact time [19] and has been considered by experts to be adequate for disinfection with liquid disinfectant products [20]. While other studies have examined the impact of contact time on the efficacy of disinfectant towelettes [21, 22], few have studied this variable under conditions reflective of realistic use. Tarka et al., (2019) [23] examined the efficacy of several disinfectant towelette products across a range of contact times; however, the products studied all had contact times of one min or less, and the contact times examined were at or beyond the label contact time for all products studied.

The objective of this study was to determine the efficacy of disinfectant towelette products when applied for a practical, relevant contact time of one min compared to contact times of both shorter and longer duration, and when done so under conditions designed to reflect realistic use. We tested five disinfectant towelette products against *S. aureus* and *P. aeruginosa* below, at, and beyond label-defined contact time using an experimental model designed to reflect realistic use. We hypothesized that contact time would significantly affect the efficacy of the disinfectant towelette products tested, and that disinfectant towelette products with longer label contact times would not achieve the desired efficacy at lower contact times (i.e., one min) that are more representative of those seen with realistic use.

## Materials and methods

Five disinfectant towelette products (Table 1) were tested against *S. aureus* (ATCC 6538) and *P. aeruginosa* (ATCC 15,442) at five contact times (30 s, one min, two min, three min, and 10 min). Three biological replicates were conducted for each disinfectant, contact time, and bacteria permutation. Experimental procedures were adapted from EPA MB-33-00 [24].

**Table 1:** Description of disinfectant towelettes

Disinfectant Products^a^	Active Ingredients^b^	Dilution at Use	Label Contact Time^d^
HP1	0.5% hydrogen peroxide	RTU^c^	1 min
QAC1	0.25% n-Alkyl (68% C_12_, 32% C_14_) dimethyl ethylbenzyl ammonium chlorides, 0.25% n-Alkyl (60% C_14_, 30% C_16_, 5% C_12_, 5% C_18_) dimethyl benzyl ammonium chlorides, 55% isopropyl alcohol)	RTU	2 min
QAC2	0.125% n-Alkyl (68% C_12_, 32% C_14_) dimethyl ethylbenzyl ammonium chlorides, 0.125% n-Alkyl (60% C_14_, 30% C_16_, 5% C_12_, 5% C_18_) dimethyl benzyl ammonium chlorides	RTU	3 min
QAC3	0.14% n-Alkyl (68% C_12_, 32% C_14_) dimethyl ethylbenzyl ammonium chlorides), 0.14% n-Alkyl (60% C_14_, 30% C_16_, 5% C_12_, 5% C_18_) dimethyl benzyl ammonium chlorides	RTU	3 min
QAC4	Octyl decyl dimethyl ammonium chloride, dioctyl dimethyl ammonium chloride, didecyl dimethyl ammonium chloride, n-Alkyl (50% C_14,_ 40% C_12_, 10% C_16_) dimethyl benzyl ammonium chloride (848 ppm active at 1:256 use-dilution)	1:256	10 min

^a^ Abbreviated naming scheme for commercially available EPA-registered disinfectants used in this study;

^b^ Concentrations of active ingredients at use-dilution;

^c^ Ready-to-use;

^d^ Recommended label contact time for standard use.

### Preparation of the test suspension

Test suspensions with soil load were prepared according to EPA MB-33-00 [24]. Briefly, 10 mL of tryptic soy broth (TSB; BD, New Jersey, USA) were inoculated with 100 µL of thawed bacterial stock and incubated at 36 ± 1 °C for 16–24 h with 200 rpm shaking. Soil components were added to the bacterial cultures to produce a test suspension containing 0.25% (w/v) bovine serum albumin (Thermo Fisher, Waltham, MA), 0.35% (w/v) yeast extract (Thermo Fisher, Waltham, MA), and 0.08% mucin (MilliporeSigma, Burlington, MA).

### Preparation of the wiping templates

**Fig. 1:**
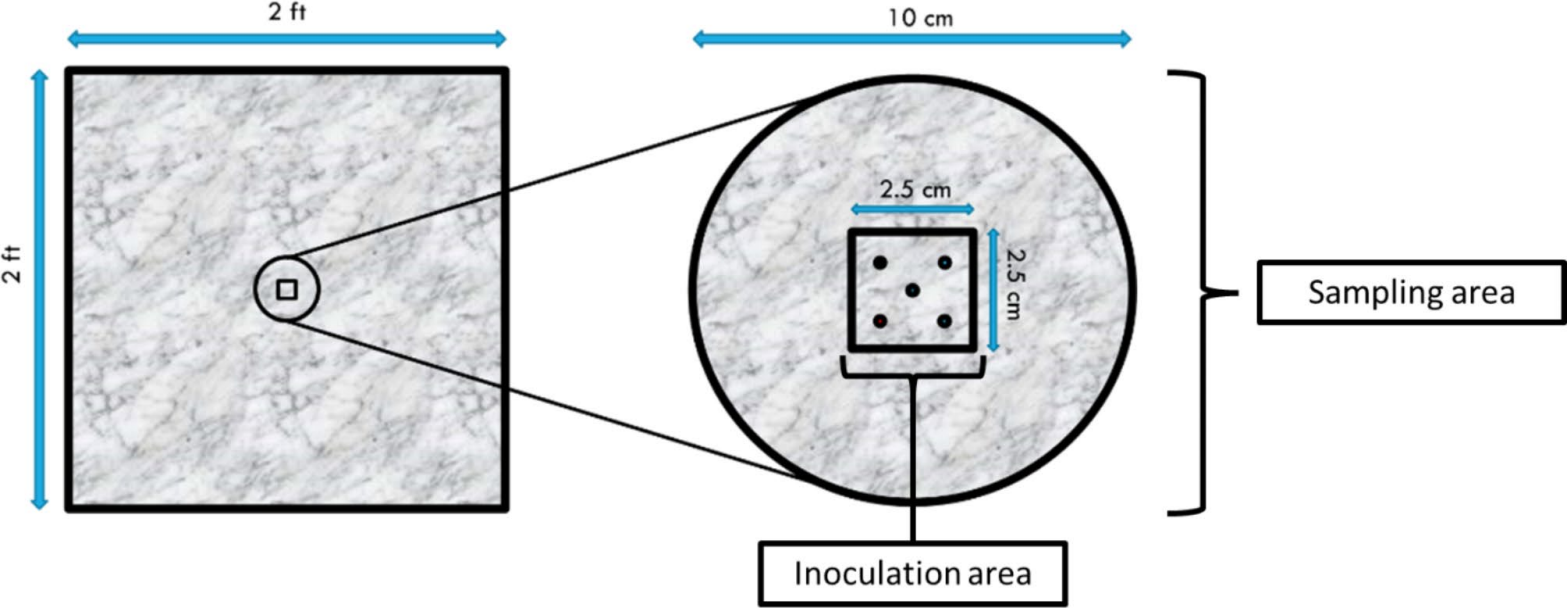
A wiping template (left) was defined as 2 ft x 2 ft in area. A 2.5 cm x 2.5 cm square was traced to serve as the inoculation area. Within this square, five droplets of 10 µL inoculum were dispensed in an “X” formation. A sampling area 10 cm in diameter was defined around the inoculation area. The sampling area was swabbed with a neutralizing buffer saturated swab to collect residual test inoculum. This design was adapted from EPA MB-33-00 [24]

Wiping templates were defined as 2 ft x 2 ft squares on Formica® laminate sheets (Midwest Manufacturing, Eau Claire, WI) (Fig. 1). Templates were disinfected prior to inoculation by applying 10% bleach, followed by 0.3% hydrogen peroxide. The board was then rinsed three times with sterile ultrapure water followed by a final application of 70% ethanol. Each template was inoculated with five 10 µL droplets of test suspension in the designated inoculation area (Fig. 1). This volume yielded an average recovery of 1.2 × 10^7^ CFU for *S. aureus* and 2.8 × 10^7^ CFU for *P. aeruginosa* upon drying. The templates were left undisturbed under ambient conditions until the inoculum was fully dried (approximately 1–2 h).

### Preparation of the disinfectant wipes

Four of the five test disinfectants (HP1, QAC1, QAC2, and QAC3; Table 1) were purchased as ready-to-use pre-saturated wipes. Prior to use, canisters were inverted several times to distribute the disinfectant throughout the canister and lids were wiped with 70% ethanol to minimize contamination during handling. Prior to testing, the first three wipes were removed from the canister to ensure that the wipes used for testing contained an even distribution of liquid disinfectant. QAC4 was purchased as a concentrate and diluted 1:256 in synthetic hard water and applied to EasyWipes (Diversey Holdings LTD, Fort Mill, SC). Synthetic hard water was prepared using the guidance for AOAC Hard Water as described in EPA MB-30-02 [25]. EasyWipes were cut to approximately 6” x 6.8”, a size comparable to that of the ready-to-use wipes used in the study. The dry wipes were impregnated on the day of use using a liquid-to-towelette ratio of 4.9 mL diluted disinfectant per wipe based on the suggested ratio provided on the EasyWipes canister.

### Disinfectant testing

**Fig. 2:**
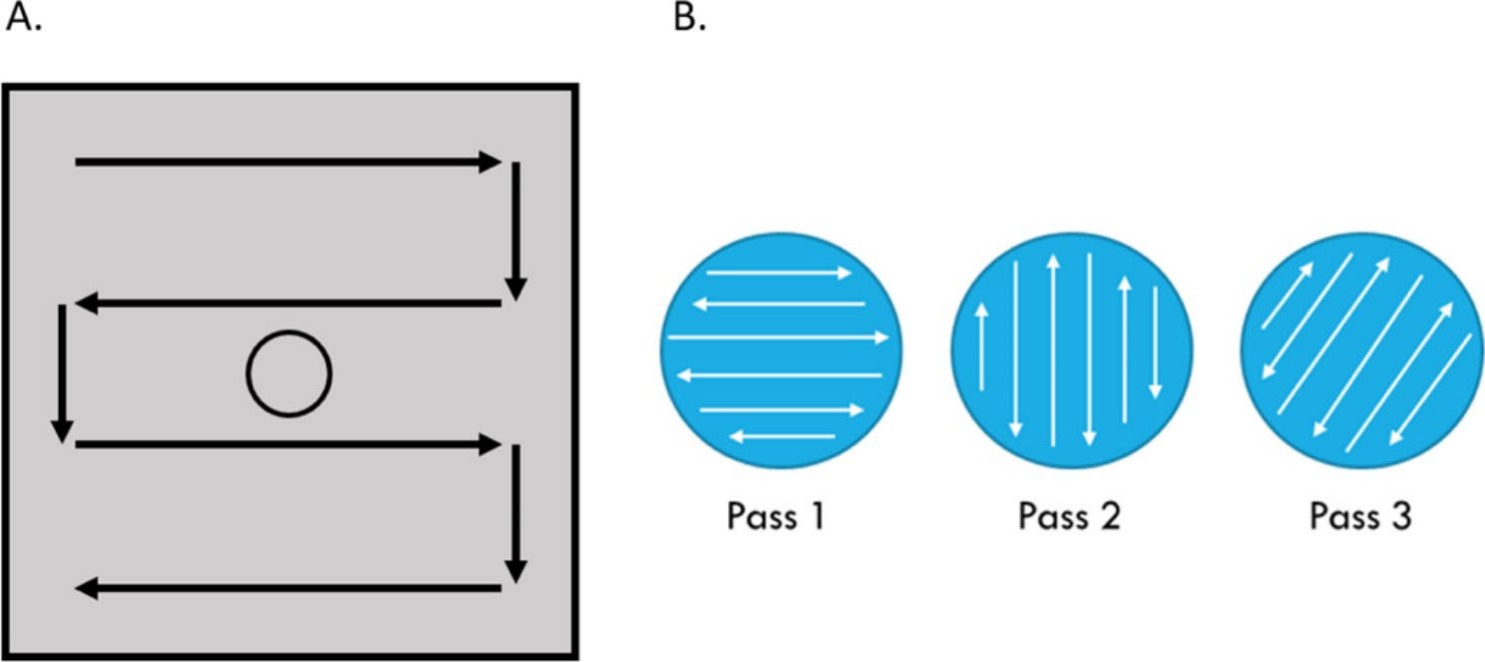
**A.** Wiping pattern on the board. Templates were wiped in four horizontal passes. Contact time for each product was set to record post-wiping. **B.** The sampling area was swabbed three times in its entirety; the first pass was done horizontally, the second pass vertically, and the third pass at a diagonal starting in the upper left and ending in the lower right of the sampling area

A disinfectant wipe was applied to the upper left-hand corner of the wiping template and moved across the template manually using four horizontal passes (Fig. 2A). This design allowed for partial depletion of the disinfectant towelette prior to wiping over the inoculation zone. Contact time was initiated at the end of the final pass. All wiping procedures were performed by the same individual to minimize variability. After the defined contact time had elapsed for each product, a PUR-Blue™ large tip neutralizing surface swab (World Bioproducts LLC, Woodinville, WA) was passed over the sampling area three times to collect the sample (Fig. 2B). The swab was vortexed for 30 s [26] prior to serial dilution of the neutralizing buffer in phosphate buffered saline. Dilutions were filtered over 0.2 μm polyethersulfone filters (Pall Corporation, Port Washington, NY) using an EZ-Fit™ Manifold filtration system (MilliporeSigma, Burlington, MA). Filters were plated onto tryptic soy agar (TSA; BD, Franklin Lakes, NJ) and incubated at 36 ± 1 °C for 24–48 h prior to counting. A non-inoculated template served as a negative control and two inoculated, untreated templates served as positive controls. Negative controls were swabbed with macrofoam swabs (World Bioproducts LLC, Woodinville, WA) saturated with sterile phosphate buffered saline and processed as described above.

### Terminal cleaning

After all templates were sampled, a terminal cleaning protocol was performed to remove residual soils prior to further testing. A 10% bleach solution was applied for a minimum of 10 min followed by 0.3% hydrogen peroxide. A multi-purpose cleaning spray (Babyganics, Westbury, NY) was applied and wiped dry with paper towels. The board was then rinsed once with ultrapure water followed by a final application of 70% ethanol.

### Data analysis

Dilutions yielding colony counts of 20–200 CFU were used for calculating bacterial recoveries. If two or more dilutions for a given test yielded a count within this range, the dilution with the higher total CFU was used. When no colonies were present across any dilutions filtered, a count of 1 CFU was used for calculations at the lowest dilution filtered. All colony counts from untreated and treated templates were log-transformed. Bacterial log reduction was calculated by subtracting the log density of a treated template from the average log density of the two control templates for a given replicate. Three biological replicates were performed for each combination of disinfectant and organism.

Statistical analyses were conducted in SAS software, Version 9 of the SAS system for Linux. Copyright© 2012–2018 SAS Institute Inc. SAS and all other SAS Institute Inc. product or service names are registered trademarks or trademarks of SAS Institute Inc., Cary, NC, USA. A general linear model was performed to determine the overall effects of contact time and product type on disinfectant efficacy for each organism studied. A Tukey’s t-test post-hoc analysis was conducted to determine differences among individual contact times and products for a given organism. Figures were prepared using Microsoft Office Suite (Microsoft Office 365) and GraphPad Prism version 9.4.1 for Mac OS X, GraphPad Software, San Diego, CA, USA.

## Results

**Fig. 3:**
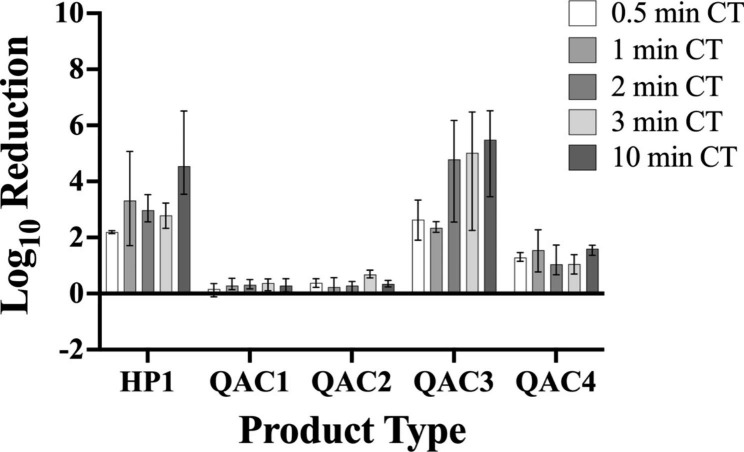
Efficacy of Disinfectant Towelette Products Against *P.aeruginosa* Mean log_10_ reduction of *P. aeruginosa* achieved by each disinfectant towelette product type at each contact time tested. Bars indicate minimum and maximum values measured

**Fig. 4:**
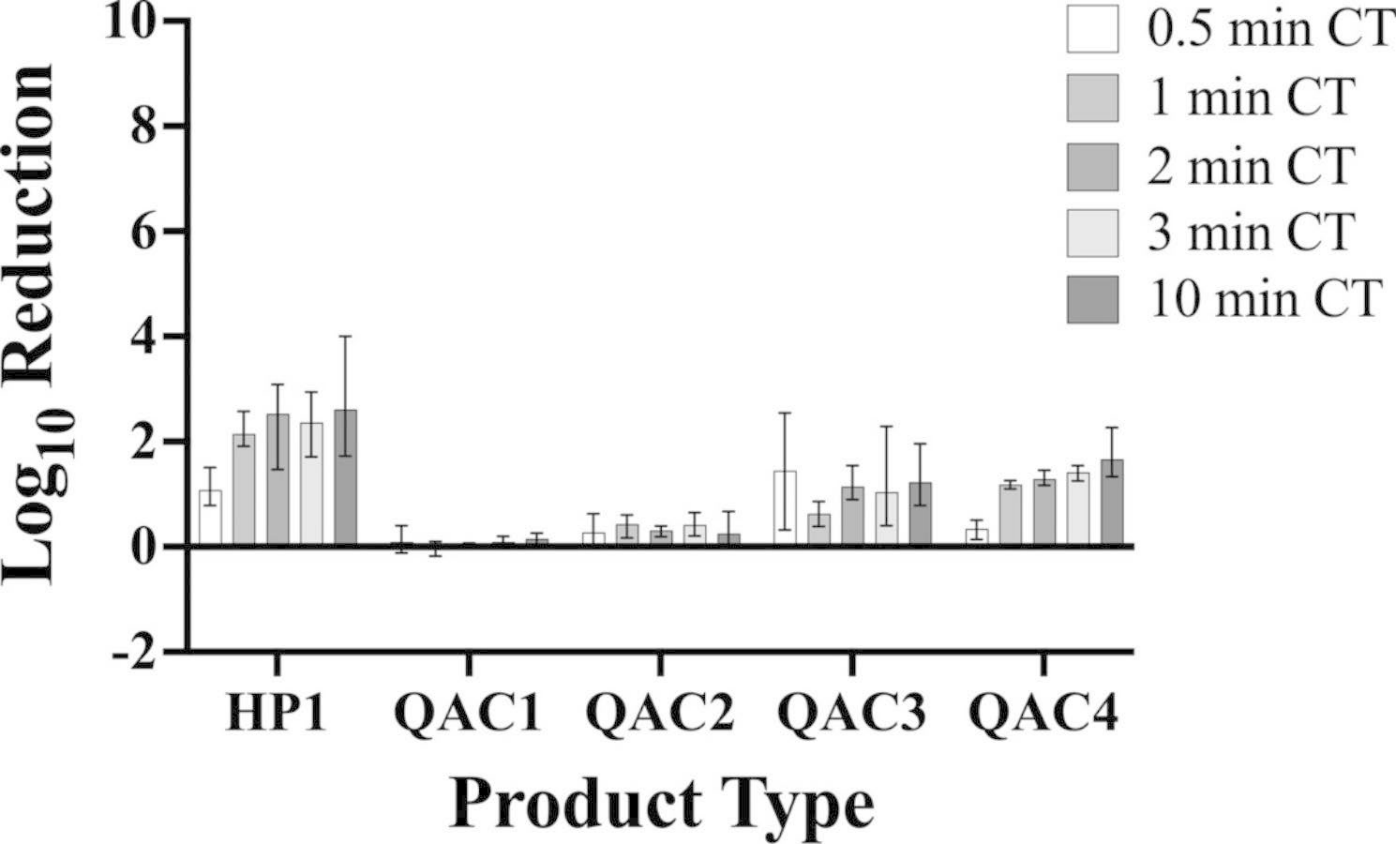
Efficacy of Disinfectant Towelette Products Against *S.aureus.* Mean log_10_ reduction of *S.aureus* achieved by each disinfectant towelette product type at each contact time tested. Bars indicate minimum and maximum values measured

Overall, we found that the efficacy of the disinfectant towelettes was significantly affected by product type, but the effect of contact time was limited. The mean log_10_ reductions for each product type at all tested contact times i.e., 30 s, one min, two min, three min, and 10 min for *P. aeruginosa* and *S. aureus* are shown in Figs. 3 and 4, respectively.

### Contact time has limited effect on bacterial log reduction

**Fig. 5:**
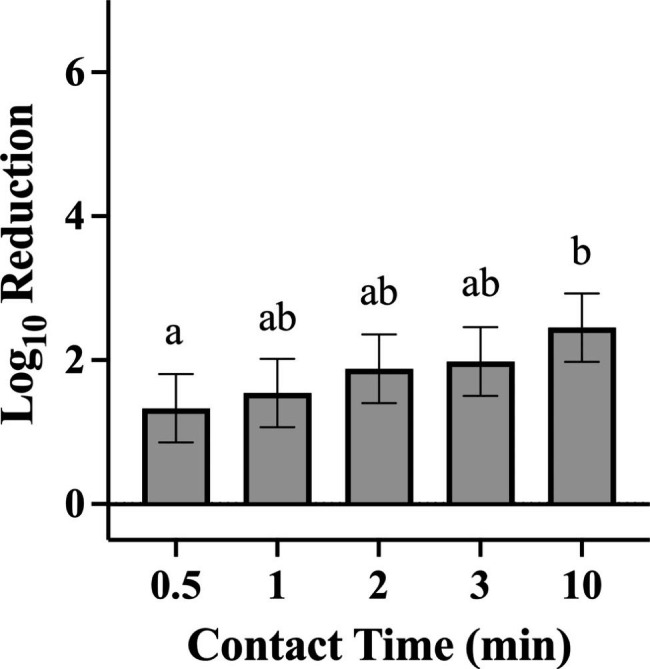
Efficacy Achieved by Disinfectant Towelette Products Against *P. aeruginosa* by Contact Time. Least Squares Means with 95% Confidence Intervals log_10_ reduction of *P. aeruginosa* achieved at each contact time tested across all products. Letters are Tukey groupings for disinfectant towelette efficacy at different contact times against *S. aureus*. Bars with the same letters are not statistically different

**Fig. 6:**
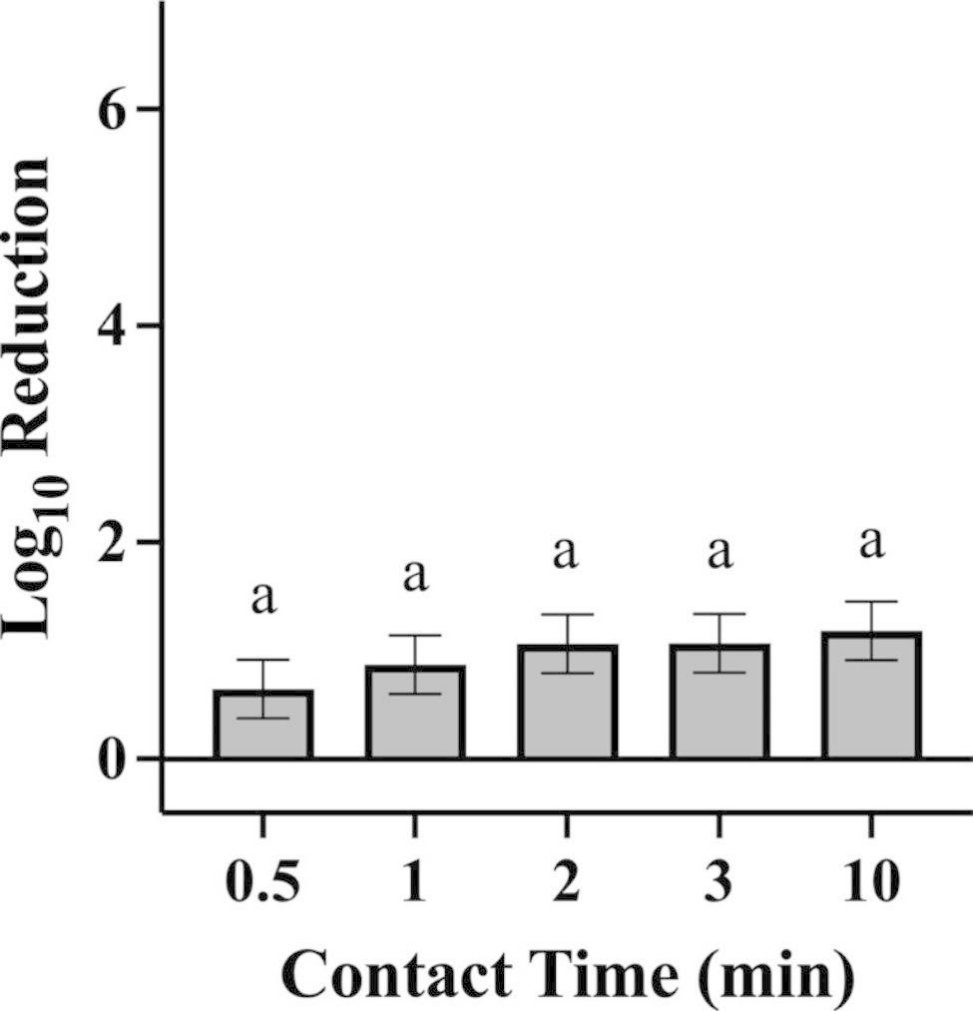
Efficacy Achieved by Disinfectant Towelette Products Against *S. aureus* by Contact Time. Least Squares Means with 95% Confidence Intervals log_10_ reduction of *S. aureus* achieved at each contact time tested across all products. Letters are Tukey groupings for disinfectant towelette efficacy at different contact times against *S. aureus*. Bars with the same letters are not statistically different

For tests against *P. aeruginosa*, a contact time of 10 min resulted in significantly higher log reductions when compared with a contact time of 30 s (Fig. 5; p = 0.0129). No significant differences among individual contact times were observed for *S. aureus***(**Fig. 6). No significant difference in bacterial log reduction was observed with a contact time of one min compared with any other contact time tested for either organism.

### Product type significantly affects bacterial log reduction

**Fig. 7:**
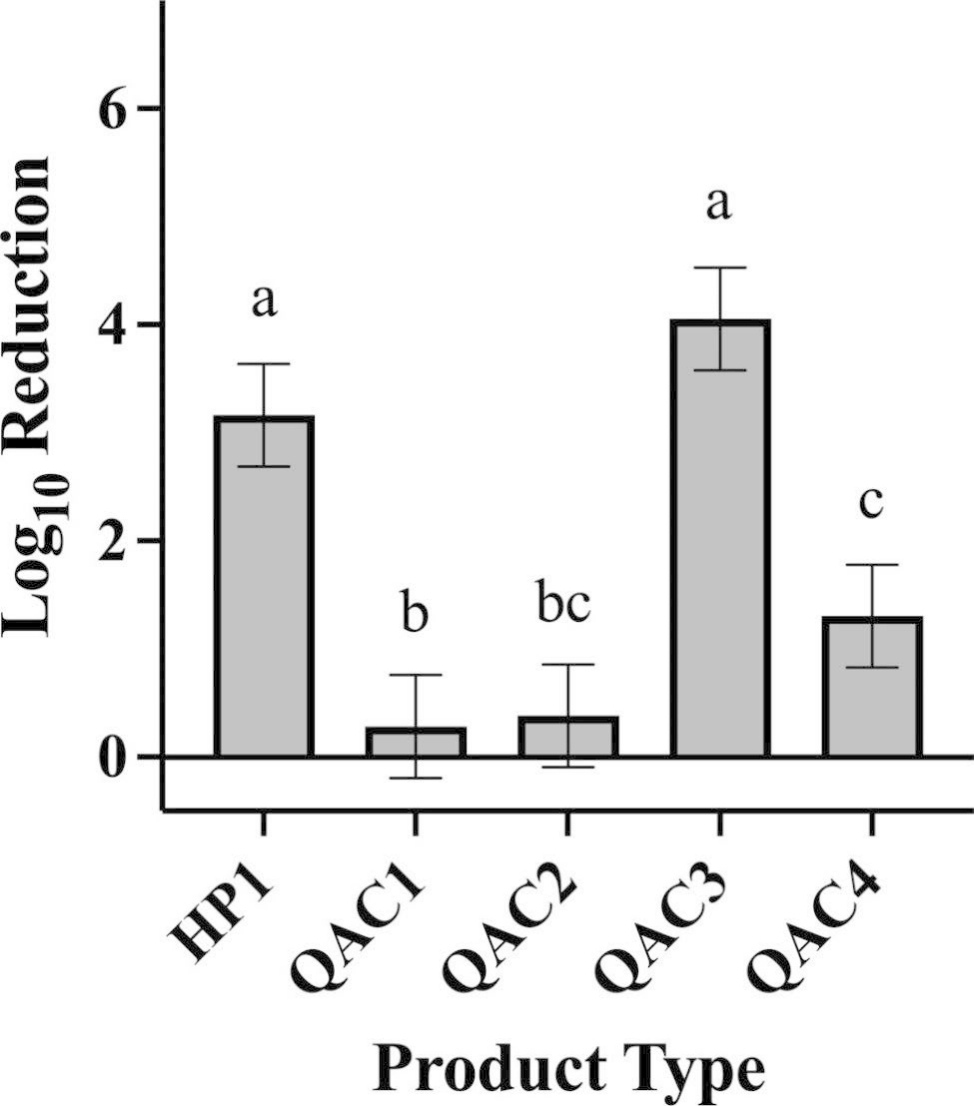
Efficacy Achieved by Disinfectant Towelette Products Against *P.aeruginosa* by Product Type. Least Squares Means with 95% Confidence Intervals log_10_ reduction of *P. aeruginosa* achieved by each product type across all contact times tested. Letters are Tukey groupings for disinfectant towelette efficacy against *P. aeruginosa*. Bars with the same letters are not statistically different

**Fig. 8:**
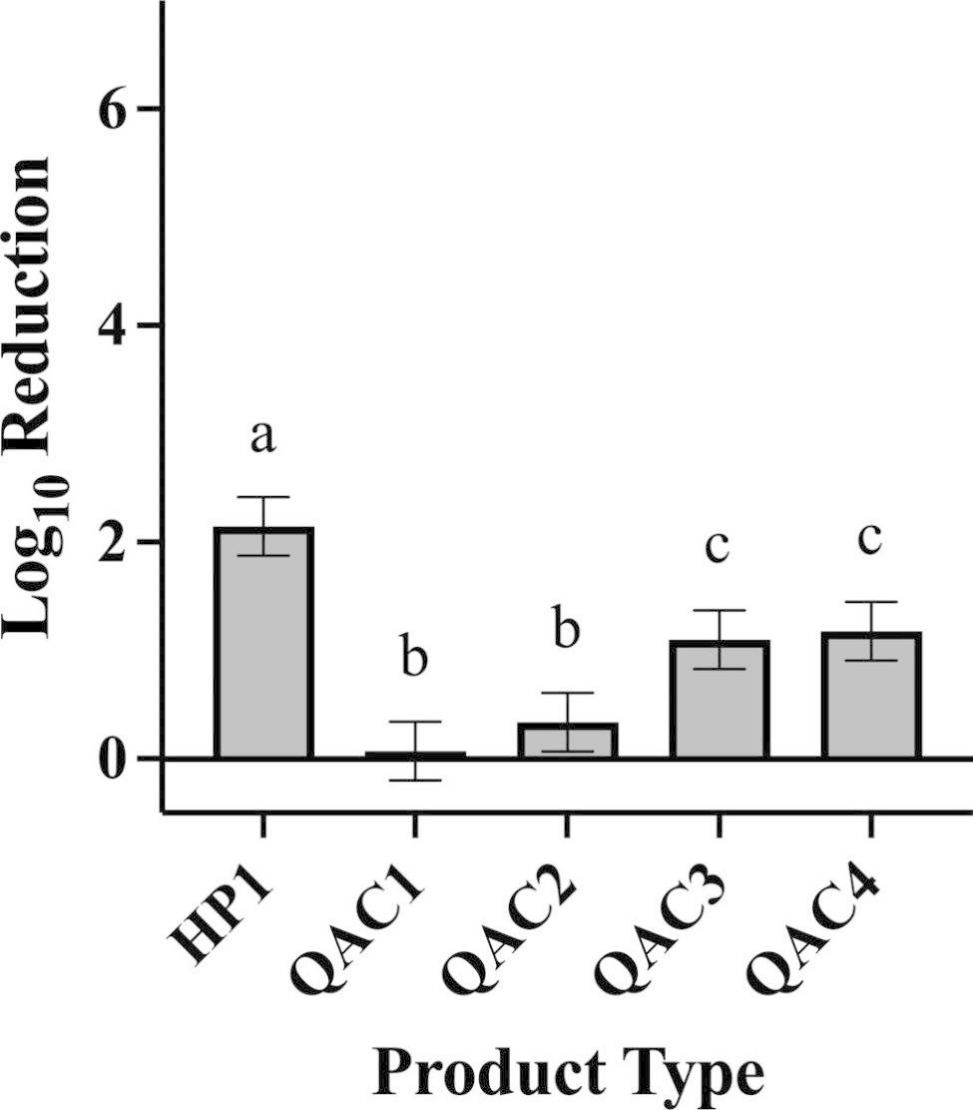
Efficacy Achieved by Disinfectant Towelette Products Against *S.aureus* by Product Type. Least Squares Means with 95% Confidence Intervals log_10_ reduction of *S. aureus* achieved by each product type across all contact times tested. Letters are Tukey groupings for disinfectant towelette efficacy against *S. aureus*. Bars with the same letters are not statistically different

In testing against *P. aeruginosa*, HP1 and QAC3 yielded greater log reductions than QAC1 (Fig. 7; p < 0.0001 for both), QAC2 (Fig. 7; p < 0.0001 for both), and QAC4 (Fig. 7; p < 0.0001 for both). QAC4 yielded significantly higher log reductions compared with QAC1 (Fig. 7; p = 0.0287). In testing against *S. aureus*, HP1 yielded a higher overall log reduction compared to all other products tested (Fig. 8; p < 0.0001 for QACs 1–4). Testing with QAC4 resulted in greater log reduction of *S. aureus* than with QAC1 (Fig. 8; p < 0.0001) and QAC2 (Fig. 8; p = 0.0005). QAC3 also yielded significantly greater log reductions than did QAC1 (Fig. 8; p < 0.0001) and QAC2 (Fig. 8; p = 0.0019).

## Discussion

The objective of this study was to determine the impact of contact time on the efficacy of disinfectant towelette products against two clinically relevant bacteria i.e., *S. aureus* and *P. aeruginosa*, under conditions designed to reflect realistic use. The results from this study indicate that the impact of contact time on the efficacy of disinfectant towelettes is limited under these conditions. When evaluating the effect of contact time on disinfectant efficacy overall, contact time did not have a significant effect on disinfectant towelette efficacy in testing against *S. aureus* when comparing the individual contact times tested. For *P. aeruginosa*, the only significant difference seen among specific contact times was for that of 30 s and 10 min, which were the two most extreme contact times tested in this study. Further, no significant differences were observed between a contact time of one min and any of the other contact times tested when examining the overall effect of contact time.

The effect of contact time was evaluated as the performance of all disinfectant towelette products against the test organism at a given timepoint. The aim of this study was to determine if a practical contact time of 1 min could generally achieve sufficient disinfection, not to evaluate the performance of individual disinfectant towelette products. Therefore, we did not evaluate the effect of contact time for each individual disinfectant towelette product.

We hypothesized that a contact time of one min would not be sufficient for products with a label contact time of longer duration. Previous research [27] demonstrated that disinfectants were significantly less effective at contact times shorter than label contact time; however, the method used for testing efficacy was different than the one used in the present study as Hong et al., (2017) [27] examined liquid disinfectants. West et al., (2018) [28] also observed a significant effect of contact time when testing liquid disinfectant. The present findings suggest that the effect of contact time is different when examining disinfectant towelette products using a method designed to reflect realistic use. Research examining the efficacy of disinfectant towelette products reports similar findings to those seen in the study presented here. West et al., (2019) [22] observed that the type of disinfectant towelette product tested against *S. aureus* significantly impacted efficacy while contact time did not. Brown et al., (2020) [21] found that the type of disinfectant used had a greater effect on disinfectant efficacy than did contact time. The discrepancy may be related to the type of disinfectant product studied, i.e., liquid disinfectants vs. disinfectant towelettes, as experts have already defined contact time differently for liquid disinfectants versus disinfectant towelette and spray products due to differences in their testing methods [29]. These findings suggest that the choice of disinfectant towelette product for disinfection may matter more than the contact time over which it is applied. Further investigation into the relative importance of different factors of disinfectant efficacy among the varied types of disinfectants is warranted.

Among the disinfectant towelette products tested, HP1, a wipe impregnated with hydrogen peroxide-based disinfectant, performed the best against both *S. aureus* and *P. aeruginosa*. Conversely, QAC1, a wipe impregnated with a quat alcohol-based disinfectant, resulted in the least log reduction of both *S. aureus* and *P. aeruginosa*. It was hypothesized that HP1 would perform best as it is the product with the shortest label contact time, and therefore, should be effective at most of the contact times tested in the study as compared to QAC4, which had the longest contact time of the products tested and was not expected be effective at contact times lower than its label contact time. However, QAC1 had the second shortest contact time of the products tested and resulted in the least log reductions of both *S. aureus* and *P. aeruginosa*. These findings further support the notion that contact time plays a limited role in disinfectant towelette efficacy.

While others have examined the efficacy of disinfectant towelettes across varied contact times, the current study is novel because the conditions under which the disinfectant towelette products were tested were designed to be more representative of realistic use, such as in a clinical setting. Under these conditions, the majority of the disinfectant towelettes did not achieve the minimum 5-log reduction required under the EPA Product Performance Test [30] Guidelines, even at their label contact times. Only QAC3 was able to achieve an average 5-log reduction under the testing conditions used in this study; even then, this was only achieved against *P. aeruginosa*, not *S. aureus.* This discrepancy in performance under the novel testing conditions is not surprising, as this has been seen previously in the literature when testing commercial disinfectant towelette products under conditions more reflective of realistic use [23, 31]. Further, others [32] have found that the method used to test disinfectant efficacy results in significant differences in performance. These results support the notion that current testing methods are not adequately reflective of the performance of these products in real life [16, 17].

The primary limitations of this study relate to variability within the study design. One such limitation is the lack of control of confounding variables associated with a model reflective of realistic use. This variability is acknowledged by others in the field [33]. The testing conditions implemented in this study model differed from the conditions used in EPA MB-33-00 [24], as the disinfectant towelettes were wiped over a larger surface made of a different material. Such factors are relevant to the disinfection process [12]. However, the individual contributions of these variables were not assessed as part of the present study. Thus, it can be hypothesized that the discrepancy in expected performance based on label claims and the actual performance of disinfectant towelette products tested in this study is explained by using testing conditions reflective of realistic use; however, it is difficult to speculate which specific conditions may be responsible for these discrepancies.

## Conclusion

Contact time had limited impact on the efficacy of several disinfectant towelette products when applied in a model designed to simulate realistic use of disinfectants such as that seen in clinical settings. Significant differences in efficacy were seen among the different product types tested, and this finding was consistent in testing against both *S. aureus* and *P. aeruginosa*. These findings suggest that the disinfectant towelette product used may have a greater impact on disinfectant efficacy than other factors such as contact time, but further research is warranted.

## Data Availability

All quantitative data generated or analyzed during this study are included in this published article.

## List of abbreviations

ATCC: American Type Culture Collection
CDC: Center for Disease Control
HAIs: Healthcare-associated infections
HP: Hydrogen Peroxide
QAC: Quaternary Ammonium Compounds
EPA: Environmental Protection Agency
